# Autosomal STRs Provide Genetic Evidence for the Hypothesis That Tai People Originate from Southern China

**DOI:** 10.1371/journal.pone.0060822

**Published:** 2013-04-08

**Authors:** Hao Sun, Chi Zhou, Xiaoqin Huang, Keqin Lin, Lei Shi, Liang Yu, Shuyuan Liu, Jiayou Chu, Zhaoqing Yang

**Affiliations:** The Department of Medical Genetics, Institute of Medical Biology, Chinese Academy of Medical Sciences & Peking Union Medical College, Yunnan, China; University of Florence, Italy

## Abstract

Tai people are widely distributed in Thailand, Laos and southwestern China and are a large population of Southeast Asia. Although most anthropologists and historians agree that modern Tai people are from southwestern China and northern Thailand, the place from which they historically migrated remains controversial. Three popular hypotheses have been proposed: northern origin hypothesis, southern origin hypothesis or an indigenous origin. We compared the genetic relationships between the Tai in China and their “siblings” to test different hypotheses by analyzing 10 autosomal microsatellites. The genetic data of 916 samples from 19 populations were analyzed in this survey. The autosomal STR data from 15 of the 19 populations came from our previous study (Lin et al., 2010). 194 samples from four additional populations were genotyped in this study: Han (Yunnan), Dai (Dehong), Dai (Yuxi) and Mongolian. The results of genetic distance comparisons, genetic structure analyses and admixture analyses all indicate that populations from northern origin hypothesis have large genetic distances and are clearly differentiated from the Tai. The simulation-based ABC analysis also indicates this. The posterior probability of the northern origin hypothesis is just 0.04 [95%CI: (0.01–0.06)]. Conversely, genetic relationships were very close between the Tai and populations from southern origin or an indigenous origin hypothesis. Simulation-based ABC analyses were also used to distinguish the southern origin hypothesis from the indigenous origin hypothesis. The results indicate that the posterior probability of the southern origin hypothesis [0.640, 95%CI: (0.524–0.757)] is greater than that of the indigenous origin hypothesis [0.324, 95%CI: (0.211–0.438)]. Therefore, we propose that the genetic evidence does not support the hypothesis of northern origin. Our genetic data indicate that the southern origin hypothesis has higher probability than the other two hypotheses statistically, suggesting that the Tai people most likely originated from southern China.

## Introduction

Tai people are a subgroup of Tai language speakers who are widely distributed in Southeast Asia and the Yunnan Province of Southwest China. Tai people are the largest ethnic group in Thailand, but this ethnic group is called different names in other countries. They are called Dai in China, Shan in Burma and Lao in Laos. Although different names are used in different countries or in different literature, most researchers agree that these Tai speakers share a recent common origin [1], [2], [3], [4], [5], [6]. For clarity, in this paper, we use “Tai” to represent the Tai speakers of Southeast Asia and Southwest China.

Even though most researchers agree that Tai people share a recent common origin, the source of the Tai migration remains controversial. There are several popular hypotheses for the place from which the Tai people came, and these hypotheses can generally be summarized into two types: an indigenous origin hypothesis [3], [7], [8], [9] and a migration hypothesis [1], [2], [5], [6], [10], [11], [12], [13]. The migration hypothesis can be further divided into migration from northern China (northern origin hypothesis) [1], [2], [10], [11] and migration from southern China (southern origin hypothesis) [5], [6], [12], [13].

The theory that the Tai originated from northern China was introduced in the late 19^th^ and early 20^th^ centuries [1], [2], [10], [11], [14]. The most important proponent was W. C. Dodd, and his theory was widely accepted by scholars of Thailand and Burma. He believed that Tai people originated from the temperate grasslands in northern China, where they lived until Chinese Han people drove them south approximately 3,000 years ago. According to this theory, Tai people were first driven to central China from the north by the Han, and then they gradually moved to parts of southwestern China, such as Yunnan and other countries in Southeast Asia, after the 6^th^ century B. C. Dodd also suggested that Tai people and Mongolians share a recent common origin [1]. The southern origin hypothesis was proposed in the early 20^th^ century by Davies [12] and has been systematically expounded by Chinese scholars, such as Fan [13] and Huang [6]. These researchers believe that Tai people came from southern China and that their ancestors are the “*Yue*” people who were indigenous people in the south of ancient China. According to their theory, Tai people and the other Tai-Kadai-speaking ethnic groups in southern China, such as the Zhuang and the Mulao, have a recent common ancestor. Consistent with this hypothesis, today’s Chinese Dai and other Tai-speaking people in other countries migrated from southern China approximately 1,000 or 2,000 years ago. In 1900, the British scholar Scott suggested that Tai people were indigenous people of southwestern China and have evolved into an independent ethnic group [7]. Some Chinese scholars have attempted to prove the theory of indigenous origin using historical records [15] and archaeological evidence [9]. These scholars proposed that Tai people should be indigenous people of southwestern China, such as the Austro-Asiatic speakers (the Wa and the Bulang in China). The supporters of the indigenous theory believe that Tai people originated from the Yunnan Province of China and north of Indochina.

**Figure 1:**
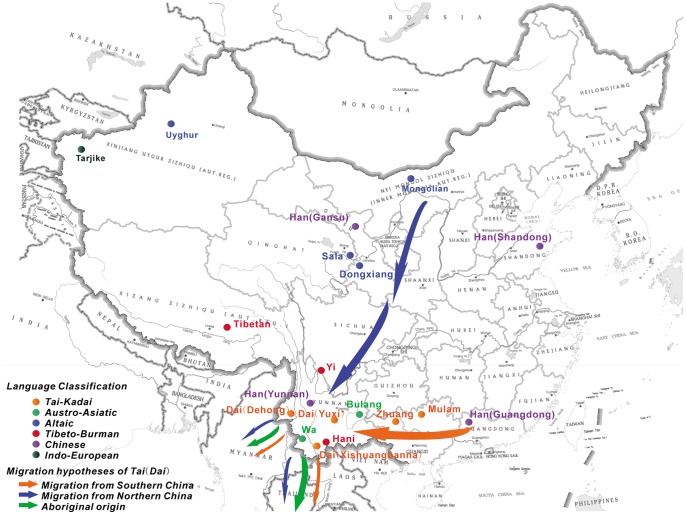
Migratory routes of Tai and populations’ information. Geographical location of the 19 sampled populations and the migratory routes from the three different hypotheses.

In addition to archeological, linguistic and historical investigations, genetic methods are also very useful for inferring some demographic events, such as population migrations, admixture and the relationships of different populations. The various theories of the origin of the Tai people were mostly derived from historical, cultural and archeological evidence but lack biological support. In this study, we use genetic methods to explore which hypothesis about the origin of the Tai is most likely correct. Nineteen ethnic groups were analyzed. Three Dai populations (Dai_Dehong, Dai_Yuxi and Dai_Xishuangbanna in Figure 1), which come from the main branches of Tai people in China, were used to represent the genetic structure of the Tai. To test the hypothesis that the Tai came from northern China, a Mongolian population was chosen. Some northern Altaic speakers (Sala and Dongxiang), who have close relationships with Mongolian people, were also chosen. We refer to the Mongolian, Sala and Dongxiang as the “northern origin of the Tai”. In the southern origin hypothesis, many Chinese ethnologists [5], [6], [13] believe that today’s Tai people migrated from southern China and that they share a recent common ancestor, which named “*Yue*” people, with other Tai-Kadai speakers who lived in southern China, such as the Zhuang and the Mulao. Thus, we refer to the Zhuang and the Mulao as the “southern origin of the Tai”. Finally, the Austro-Asiatic speakers, such as the Wa and the Bulang, were thought to share a recent common ancestor with Tai people in the indigenous origin hypothesis [8], [9]. In our study, they are called the “native origin of the Tai”.

The other six populations, including three different Han populations and three populations (Tibetan, Hani, Yi) that speak Tibeto-Burman, were chosen to test the reliability of our method based on analyses of genetic variation. There is evidence that Tibeto-Burman speakers and Han people share a recent common ancestor [16], [17] and that the Han (Yunnan) migrated to southern China hundreds of years ago; this migration was recorded in detail. The migration of the Han (Yunnan) is similar to that of Tai people. If our genetic analyses are reliable, the results should reflect this historical event. Two populations (Uyghur, Tarjike) that live near Central Asia were also chosen because there is a possibility that the current Altaic-speaking populations admixed with later arrivers from Central Asia [18]. The migratory routes of the three hypotheses are shown in Figure 1. All of these 19 populations’ geographic locations and their language classifications are shown in Figure 1 and Table 1.

**Table 1:** Details from the 19 sampled populations.

No.	Population	Size	Location	Lat(N)	Long(E)	Language classification
1	Dai (Xishuangbanna)	60	Xishuangbanna, Yunnan	22.01	100.79	Tai-Kadai, Kam-Tai
2	Dai (Dehong)*	48	Dehong, Yunnan	24.25	98.27	Tai-Kadai, Kam-Tai
3	Dai (Yuxi)*	48	Yuxi, Yunnan	24.07	101.99	Tai-Kadai, Kam-Tai
4	Zhuang	56	Baise, Guangxi	23.90	106.62	Tai-Kadai, Kam-Tai
5	Mulam	52	Luocheng, Guangxi	24.91	108.84	Tai-Kadai, Kam-Tai
6	Wa	49	Ximeng, Yunnan	22.64	99.60	Austro-Asiatic, Mon-Khmer
7	Bulang	39	Luxi, Yunnan	23.44	98.59	Austro-Asiatic, Mon-Khmer
8	Sala	53	Xunhua, Qinghai	35.87	102.43	Altaic, Turkic
9	Uyghur	42	Yili, Xinjiang	43.92	81.32	Altaic, Turkic
10	Mongolian*	50	Damaoqi, Inner Mongolia	41.70	110.43	Altaic, Mongolic
11	Dongxiang	45	Dongxiang, Gansu	35.66	103.39	Altaic, Mongolic
12	Tibetan	46	Lhasa, Tibetan	29.66	91.13	Tibeto-Burman, Himalayish
13	Yi	62	Ninglang, Yunnan	27.28	100.75	Tibeto-Burman, Burmic
14	Hani	55	Jinghong, Yunnan	22.01	100.79	Tibeto-Burman, Burmic
15	Han (Yunnan)*	48	Midu, Yunnan	25.33	100.47	Chinese
16	Han (Gansu)	39	Weiwu, Gansu	37.92	102.63	Chinese
17	Han (Guangdong)	39	Guangning, Guangdong	23.63	112.44	Chinese
18	Han (Shandong)	45	Zouping, Shandong	36.86	117.74	Chinese
19	Tarjike	40	Tashikuergan, Xinjiang	37.77	75.23	Indo-European, Indo-Iranian
	Total	916				

Lat and Long represent latitude (north) and longitude (east), respectively.

An *indicates that the 10 STR genotyping was performed in this study.

Microsatellites are highly informative loci that are found across the human genome that can provide generally reliable relationships among closely related populations [19]. Compared to mitochondrial DNA (mtDNA) and variations of the Y chromosome, autosomal microsatellites carry the genetic information of both parents. Thus, we chose microsatellites to trace the migratory routes of the Tai people. Genetic data of 916 samples from 19 populations were analyzed in this study. Genotype data for 15 of the populations came from our previous study (Lin et al., 2010) [20]. 194 samples from four additional populations were genotyped in this study. The four populations were Han (Yunnan), Dai (Dehong), Dai (Yuxi) and Mongolian. To compare the new data with previously reported data, 10 microsatellites that were used by Lin [20] were also used in this study.

## Results

### Standard Diversity Indices

All of the 19 populations, which included 916 samples, were analyzed. Four of the populations were genotyped in this study, and the data from the other 15 populations is from Lin’s study [20]. The amount of missing data was less than 5% in each population. Expected heterozygosities (*H*_E_) [21] were used to describe the polymorphisms of the microsatellites from different populations, and Hardy-Weinberg Equilibrium (HWE) tests were also performed. The allele frequencies, the *H*_E_ results and HWE test results of the four populations that were genotyped in this study are shown in Table S1. The lowest *H*_E_ was 0.666 (D3S1285 of the Mongolian population), and the highest *H*_E_ was 0.898 (D3S1263 of the Yunnan (Han) population). All 10 of the markers were highly diverse in these four populations. The genotypic data of these 10 markers in 19 populations were listed in Table S2.

Most loci were consistent with HWE in the four populations that were genotyped in this study. After a Bonferroni correction for multiple tests, only two cases, D3S1278 and D3s1292 in the Han (Yunnan) population, remained significant at an α = 0.000125 significance level. This may be caused by the deficiency of heterozygotes, which could be attributed to a null allele.

Because all of the 10 markers were located on chromosome 3, although the distances between them were very far (e.g., an average of 23.5 cM for next-to-adjacent loci), analyses of linkage disequilibrium between adjacent loci were performed to ascertain that these loci were not linked. In all of the 19 populations, no significant linkage disequilibrium was identified between these loci after a Bonferroni correction was performed. Linkage disequilibrium beyond adjacent loci was not tested because the high recombination fraction between loci made linkage disequilibrium unlikely.

### *F*_ST_ and *PCA*

The fixation index *F*_ST_ [22] was calculated to measure the differentiation between populations. The results of pairwise *F*_ST_ comparisons between all 19 of the populations are shown in the lower triangle of Table S3, and their *P* values are shown in the upper triangle. The values of pairwise *F*_ST_ ranged from 0.003 to 0.071. The differences, indicated by *F*_ST_, between most of the populations were significant, even when the significance level was set at 0.0003 (after a Bonferroni correction). The *F*_ST_ value was very small and not significant among the Dongxiang, Sala and Han (Gansu) populations. The *F*_ST_ value between the Zhuang and the Mulam was also small and not significant. All of the *F*_ST_ values between the Mongolian population and the three Dai populations were greater than the *F*_ST_ value between the Zhuang and the three Dai populations.

According to the three hypotheses of the origin of the Tai, we pooled populations that are located in each of the hypothetical origins of the Tai and calculated the *F*_ST_ values among them. The Mongolian, Dongxiang and Sala populations were pooled as the NT (Northern origin of the Tai). The Zhuang and the Mulao populations were pooled as the ST (Southern origin of the Tai). The Wa and Bulang populations were pooled as the NAT (Native origin of the Tai). The three Dai populations were pooled as the Tai of China (TC).

The *F*_ST_ value between the TC and the NT was 0.0183; the *F*_ST_ value between the TC and the ST was 0.0055; the *F*_ST_ value between the TC and the NAT was 0.0052. The differences that are indicated by the pairwise *F*_ST_ among these four pooled populations were significant (*P*<0.0001). The genetic distance between the TC and the NT was nearly three times as large as the distances between the TC and the other two pooled populations (the ST and the NAT).

**Figure 2:**
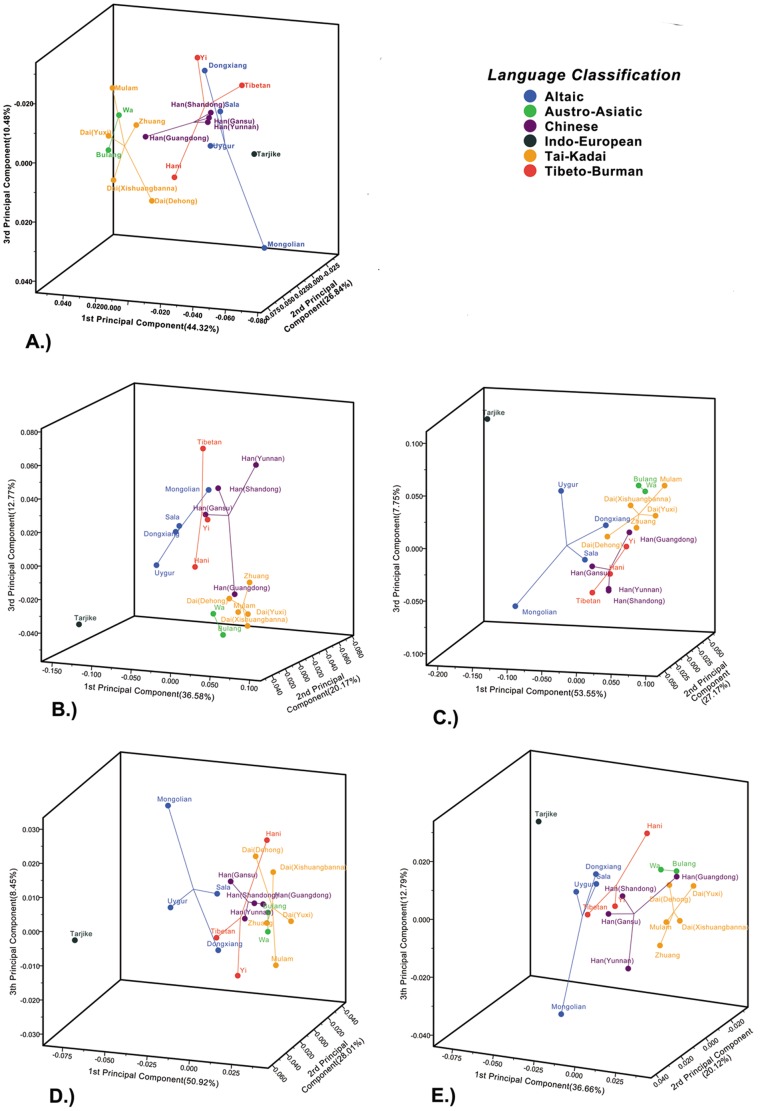
Results of PCA. Principal coordinate analyses (PCA) were performed according to A.) *F*_ST_, B.) *D_A_*, C.) *D*_ST_, D.) Latter’s *F*_ST_ * distance and E.) *D_C_*. Percentages of variance accounted for by the three components are indicated in the labels. Populations are colored according to their linguistic affiliations for better visual comparison.

In order to avoid deviations which may be caused by the difference of the distance calculation methods and catch the common features of populations’ relationships which were revealed by different genetic distances, four other genetic distances were calculated. Those are Cavalli-Sforza's chord measure (*D_C_*) [23], Nei’s distance (*D_A_*) [24], Nei’s standard genetic distance (*D*_ST_) [25] and Latter’s *F*_ST_ * distance [26]. Principal coordinate analysis (PCA) was performed to visualize the patterns of the genetic relationships based on these four genetic distances and *F*_ST_. The results are shown in Figure 2. It revealed similar genetic relationships among the 19 populations with *F*_ST_ analyses. The genetic differentiation was high between the Tai and populations from the northern origin hypothesis. Conversely, genetic differentiation was low among populations from the southern origin hypothesis or populations from the indigenous hypothesis and the Tai. In most of the PCA analyses, the Han (Yunnan, Shangdong, Gansu) clustered together and were near the populations from northern China. The Han (Guangdong) was near the populations of southern China.

### Clustering Analysis using STRUCTURE

**Figure 3:**
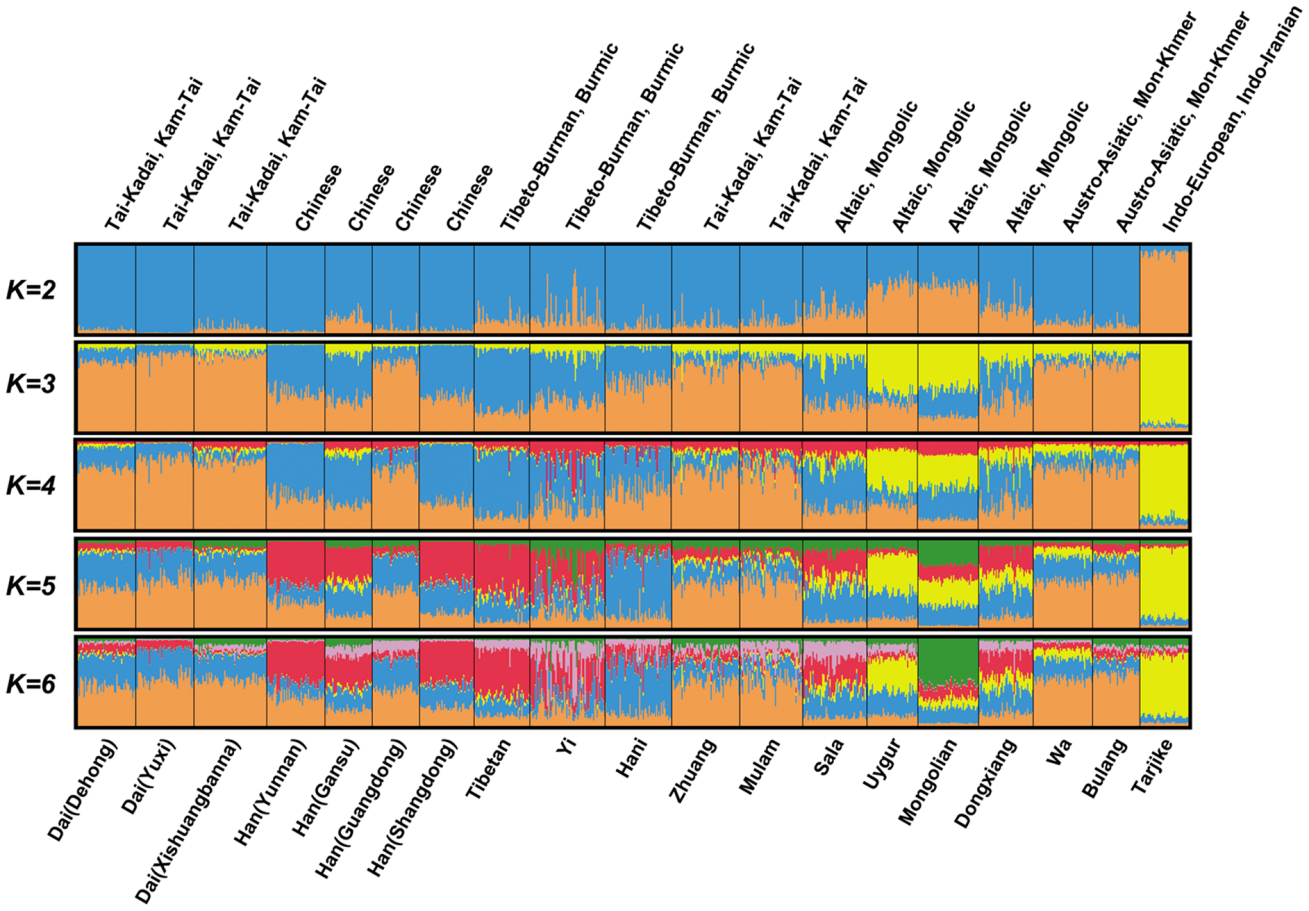
Clustering analysis from STRUCTURE. Population structure was investigated using STRUCTURE software assuming *K* = 2, 3, 4, 5, 6. Populations were ordered according to their linguistic affiliations. Linguistic affiliations and population names are labeled above and beneath the plot. According to the guidelines in the STRUCTURE manual, *K* = 5 is the most appropriate value.

Population structure was also investigated using STRUCTURE 2.3 under the LOCPRIOR model [27]. The *r*_MAX_ = 0.66±0.05 (*K* = 9) in our analyses indicates that more information is given with the LOCPRIOR model than without it. The results for *K* = 2 to 6 are shown in Figure 3. We observed a plateau of the estimated posterior probability at *K* = 5. According to the guidelines in the STRUCTURE manual [27], *K* = 5 is the most appropriate value.

At *K* = 5, the five different genetic components had similar proportions in the three Tai populations. Simultaneously, similar proportions could also be found in populations from the southern origin hypothesis (Zhuang, Mulam) and populations from the indigenous hypothesis (Wa, Bulang). A clear distinction was observed between the Tai and populations from the northern origin hypothesis (Mongolian, Sala, Dongxiang). In particular, the genetic component represented by orange (In Figure 3) prevalent in the Tai was greatly reduced in the Mongolian, Sala and Dongxiang populations. The four Han populations were divided into two clusters. The Yunnan, Shangdong and Gansu Han clustered together, and the Guangdong Han was separate from these. The genetic relationships revealed by STRUCTURE were consistent with the PCA results.

### Admixture Estimation

Because gene exchange between the Tai and native populations in southwestern China (Wa and Bulang) possibly existed after the Tai people migrated to southwestern China, it is necessary to evaluate the genetic influence of the Wa and the Bulang on the Tai people when examining both the northern and southern origin hypotheses. Admixture analyses of multi-parental populations were performed using the Admix 2.0 program [28]. The analysis parameters are listed in the Materials and Methods section. The populations were pooled in an identical manner to the way they were pooled in the Fst analyses; the Mongolian, Sala and Dongxiang populations were pooled (NT) as one parent of Tai people in China (TC); the Wa and Bulang populations were pooled (NAT) as the other parent of the TC. The estimated admixture proportion (*mY*) of the NT to the TC was −0.08±0.21 (mean±S.D.), and the estimated *mY* of the NAT to the TC was 1.08±0.21. This indicates that the NT postulated as the parents of the TC have made very little genetic contribution to the TC. According to the southern origin hypothesis, the Mulam and the Zhuang were pooled (ST) as the southern parent of the TC. The *mY* of the ST to the TC was 0.35±0.12, and the *mY* of the NAT to the TC was 0.65±0.12. Thus, the ST contributed approximately 35% of the genes to the gene pool of the TC, and the other 65% of the genes in the gene pool of the TC came from the NAT populations. Based on these results, the southern origin hypothesis appears more reasonable than the northern origin hypothesis when the gene flow after Tai migrated to southwestern China was considered.

### Approximate Bayesian Computation Testing the Model of the Origin of the Tai

**Figure 4:**
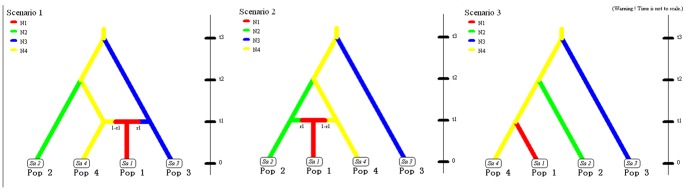
Alternative scenarios for ABC analysis. Alternative scenarios to describe the origin of the Tai for ABC analysis. Scenario 1 corresponds to the northern origin hypothesis, scenario 2 corresponds to the southern origin hypothesis and scenario 3 corresponds to the indigenous origin hypothesis. Details of the parameters used each scenario are provided in the Materials and Methods section.

The above analyses indicate that there is a large genetic distance between the NT and the Tai; therefore, the northern origin hypothesis is not supported by our genetic data. However, we still cannot distinguish whether the southern origin hypothesis or the native origin hypothesis is closer to the actual situation. These three hypotheses were simulated, and their posterior probabilities were calculated using DIY-ABC v1.0.4.46b [29], which is based on approximate Bayesian computation (ABC). Three scenarios were constructed, and the demographic parameters of these were estimated according to the three hypotheses of the origin of the Tai (Materials and Methods, Figure 4).

After generating 1.5 million simulated datasets (0.5 million for each scenario), the posterior probability of each scenario was evaluated using polychotomous logistic regression [29], [30] on the 1% of simulated datasets that were closest to the observed dataset (Table 2). The highest posterior probability was 0.64 [95%CI: (0.52–0.76)] in scenario 2, which was constructed according to the southern origin hypothesis.

**Table 2:** Model choice and performance of the ABC analysis.

Scenario	Relative posterior probability (95% CI)	P(SC2)*	Hypotheses of the origin of the Tai.
SC1	0.036 (0.008–0.063)	0.028‡	Hypothesis of Northern origin
SC2	0.640 (0.524–0.757)	0.842§	Hypothesis of Southern origin
SC3	0.324 (0.211–0.438)	0.094‡	Hypothesis of Indigenous origin

* P(SC2) is the proportion of pseudo-observed simulated datasets using each competing scenario (SC1 to SC3) for which SC2 was selected because it had the highest posterior probability.

‡ For SC1 and SC3, P(SC2) represents an empirical estimate of the model-specific type II error rate (here, 2.8%+9.4% = 12.2%).

§ For SC2, 1 − P(SC2) provides an empirical estimate of the type I error rate (here, 15.8%).

Following the recommendations of Robert et al. [31], we evaluated the power of the model choice procedure using the method implemented in DIY-ABC (Table 2). For this, we first simulated 500 random datasets under the selected scenario (scenario 2) and then computed the proportion of cases in which this scenario did not have the highest posterior probability among all of the scenarios. The empirical estimate of type I error was only 15.8%. We then empirically estimated the type II error rate by simulating 1,000 random datasets (500 for each) under the other two scenarios (scenarios 1 and 3) and computing the proportion of cases in which scenario 2 was incorrectly selected as the most likely scenario from these simulated datasets (Table 2). The average type II error rate was 12.2%, indicating a statistical power of 87.8%. Hence, this simulation-based evaluation of the performance of the ABC model choice procedure [31] clearly indicated that, given the size and polymorphism of our dataset, the method had high statistical power to distinguish between the alternative demographic scenarios that we investigated.

## Discussion

Genetic data from living human populations can be used to reconstruct their evolutionary histories and to investigate certain demographic events. Our study aimed to determine the origin of modern Tai-speaking people using genetic variation analyses. To ensure that our methods were applicable, we introduced the Han (Yunnan) into our study, the origin of which is well documented by ancient historical records. At approximately 200 B. C., tens of thousands of migrants entered Yunnan from the kingdom of *Chu*, which was located in what is today the Hubei Province of China. The largest wave of immigration to Yunnan occurred early in the Ming Dynasty in 1389 A. D. Approximately 3 million Han people, most of whom came from Nanjing (Jiangsu Province today), and a few from Shanxi and Hebei Provinces, were sent to Yunnan by the emperor of the Ming Dynasty. Thus, they all came from northern China. In Midu County, Yunnan Province, where we collected the samples representing the Han (Yunnan) population, the Han people consider themselves “pure Han people”. According to the county annals of Midu, they were the first migrants, following the army, and came from Jiangsu Province (North of the Yangtze River) in the Ming Dynasty. Actually, most of the Han people in Central Yunnan came from northern China. The spoken languages serve as further evidence indicating that the Han in Yunnan came from northern China. Today, the languages used by most Yunnan Han people belong to the northern dialect of Chinese.

The results of our genetic analyses are consistent with these historical records. The pairwise *F*_ST_ values between the Yunnan Han and the other two northern Han populations (Shangdong and Gansu) were very small (0.006 and 0.008). Furthermore, there were no statistically significant differences between them after Bonferroni correction (*P*>0.0003). Conversely, the pairwise *F*_ST_ value between the Yunnan Han and the Guandong Han, which is a typical southern Han population, was 0.018, and the difference was significant. The PCA results based on different genetic distances also indicate that the Han (Yunnan) have a closer genetic relationship to the northern Han than the southern Han. Using the southern Han population for comparison, STRUCTURE analysis also revealed more consistent proportions of genetic components in the Han (Yunnan) and other northern Han populations. Consequently, for a relatively short time, the genetic variation of the Han (Yunnan) was mainly determined by their origin, and the Han (Yunnan) and the Han in northern China still have a similar genetic makeup, although they are separated by large geographic distances. The consistency between the historical records and our genetic analyses also shows that our microsatellite-based methods can be used to correctly trace population origins. Thus, we used these methods to test the hypotheses of the origin of the Tai.

Three main hypotheses about the origin of Tai-speaking people were proposed based on archaeological and anthropological data: the indigenous origin hypothesis [3], [7], [8], [9], the northern origin hypothesis [1], [2], [10], [11] and the southern origin hypothesis [5], [6], [12], [13]. Our genetic analyses do not support the hypothesis that Tai-speaking people came from northern China. The populations that are putatively similar to the Tai from the northern origin hypothesis all have large genetic distances with the three Tai populations, as shown by the *F*_ST_ and PCA results. In particular, the Mongolian population, which was considered “near in blood with Tai people” by Dodd [1], has the second largest genetic distance to the Tai in all of the tested populations. It is difficult to explain the large genetic distance between Tai people and Mongolian people if the northern origin hypothesis is true. The genetic makeup of populations from the northern origin hypothesis, as revealed by the STRUCTURE analysis, also has obviously different patterns from those of the Tai. However, the proportion of genetic components in the Tai is very similar to those in the populations from the southern origin hypothesis (Zhuang and Mulam) and the populations from the indigenous hypothesis (Wa and Bulang). The results of the admixture analyses do not support that Tai-speaking people came from northern China. The admixture analyses for pooled populations returned a negative *mY*. This indicates that the NT did not genetically contribute to the gene pool of the TC. After immigration, a gene pool may primarily be affected by genetic drift, mutation or gene flow. Within such a relatively short period, approximately 2,000–3,000 years, it is difficult to believe that random genetic drift, mutation or gene flow from native populations (Wa and Bulang) could generate such significant differences between the Tai and populations from the northern origin hypothesis (Dongxiang, Sala and Mongolian). The simulation-based ABC analysis also indicates this. The posterior probability of the northern origin hypothesis was only 0.04 [95%CI: (0.01–0.06)]. Furthermore, certain populations that are separated by small geographic distances from populations from the indigenous hypothesis (Wa and Bulang), such as Hani and Han (Yunnan), do not share a similar genetic makeup with them. Therefore, we propose that the hypothesis of a northern China origin is unlikely.

Other reports have also shown that Tai-speaking people have large genetic distances from Mongolian populations. Yao et al. (2002) examined hypervariable segment I (HVSI) of mtDNA from several Chinese populations, including Tai-speaking people and Mongolian populations. The phylogenetic trees they constructed showed that Tai-speaking people (including Chinese Dai and Thai in Thailand) and Zhuang clustered on the tree, whereas the Mongolian population was located distantly from these populations. PCA results also showed that Mongolian people have a large genetic distance with Tai-speaking people [32]. Jin et al. (2009) sequenced HVSI and HVSII of mtDNA and reanalyzed the published Y chromosome data. In their multidimensional scaling (MDS) plot based on the mtDNA and Y haplogroups, the Mongolian population and the Tai do not cluster together; the Mongolian population is within the northern cluster, and the Tai are within the southern cluster [33]. In 2010, Stoneking and Delfin reviewed the Y chromosome haplogroup data of East Asia [34]. The O haplogroup of the Y chromosome, which is widespread in southern East Asia, prevails in the gene pools of the Tai-speaking people. In contrast, the frequencies of C haplogroups, which prevail in the Mongolian population, are very low in Tai-speaking people [33], [34]. Therefore, based on either maternal mtDNA or paternal Y chromosome genetic data, Tai-speaking people have clear genetic differences from Mongolian people. Conversely, their genetic components are very similar to the southern populations of China, which is consistent with our findings based on the analysis of autosomal microsatellites. Thus, it appears that the hypothesis that Tai people migrated from northern China cannot be supported by genetic evidence.

We excluded the northern origin hypothesis, but it is difficult to separate the southern origin hypothesis from the indigenous hypothesis. Populations from the southern origin hypothesis (Zhuang and Mulao) and those from the indigenous hypothesis (Wa and Bulang) are very closely related. The results of pairwise *F*_ST_ analysis and PCA comparisons indicate that the genetic differences among these populations are small. Furthermore, the clustering analysis from STRUCTURE also indicates that these populations are genetically similar. We also found that the mean number of different alleles per locus in the ST (12.1±1.7) was slightly larger than in the Tai (11.4±0.1) and in the NAT (10.5±1.03), but this difference was not statistically significant (Mann-Whitney U-test, *P*>0.05). To find the most likely model of the Tai origin, we inferred the possibilities of the three hypotheses of the Tai origin using the ABC approach [35] implemented in DIY-ABC v1.0.4.46b [29]. We found that the southern origin hypothesis had the highest posterior probability, specifically, 0.64 [95%CI: (0.52–0.76)], while the posterior probability of the indigenous hypothesis was 0.32 [95%CI: (0.21–0.44)]. This indicates that the southern origin hypothesis is statistically more similar to the actual situation.

Finally, in our study, we only selected Tai populations found within China, which is based on an inference agreed upon by most anthropologists and historians: modern Tai-speaking people have a recent common origin [1], [2], [3], [4], [5], [6], [36]. In fact, the genetic variation within different Tai populations is different. Even in the three Dai populations that we chose, genetic differences can be detected among them. To reveal the true migration path of Tai-speaking people, the genetic relationships among Tai-speaking people are worthy of further investigation.

## Materials and Methods

### Sampled Populations and DNA Preparation

The autosomal STR data of 15 populations came from our previous study [20]. Four additional ethnic groups were added in this study: Han (Yunnan), Dai (Dehong), Dai (Yuxi) and Mongolian. The locations and sample sizes of all of the 19 populations are listed in Table 1 and Figure 1. Samples were collected through a coordinated effort of several institutes participating in the Chinese Human Genome Diversity Project [37]. All blood contributors’ ethnic features were confirmed by tracing their family history back at least three generations and by confirming that their clans have lived in the area more than 100 years.

The DNA was obtained from immortalized B lymphocyte cell lines in our ethnic cell bank. Written informed consent was given for the establishment of cell lines and for subsequent genotyping studies. This study was approved by the Ethics Committee at the Chinese Academy of Medical Sciences and Peking Union Medical College. The DNA was extracted using a DNA Miniprep Kit (Axygen, Hangzhou, Jiangsu, China).

### Genotyping of the 10 Microsatellites

To compare our results to the published STR data [20], we chose 10 microsatellites on chromosome 3 for genotyping. These markers were taken from the ABI Prism Linkage Mapping Set. All of the markers are CA repeats, and the mean distance of adjacent markers is 23.5 cM, which is ideal for minimizing the effect of linkage disequilibrium on genetic distance calculations.

PCR reactions contained 0.2 µmol/L of primer mix, 200 µmol/L of each deoxynucleotide triphosphate (dNTP), 1×PCR Buffer, 0.1 U Transtart DNA polymerase (Transgen, Peking, China) and 20 ng of genomic DNA in a total volume of 25 µL. Amplification reactions were performed using a Perkin Elmer GeneAmp PCR System 9600 thermal cycler (Applied Biosystems, Carlsbad, California, USA), and cycling conditions were as follows: initial denaturation at 94°C for 5 min, followed by 10 cycles of 94°C for 30 s, 55°C for 30 s and 72°C for 30 s, 25 cycles of 89°C for 30 s, 55°C for 30 s and 72°C for 30 s and a final extension at 72°C for 20 min. The amplified products were detected with an ABI3730 genetic analyzer (Applied Biosystems, Carlsbad, California, USA). The data files were generated by ABI PRISM GeneScan Analysis software (Applied Biosystems, Carlsbad, California, USA).

### Data Analysis

Arlequin version 3.11 [38] was used to calculate *H*_E_ values [21] and to perform the likelihood ratio test to verify the lack of linkage disequilibrium [39] between adjacent markers. HWE tests were performed by the Genepop [40] program for every population and locus. Arlequin was used to estimate *F*_ST_ values [22] between paired populations, and their significances were tested with 50,000 permutations. Significance levels for these values were adjusted by sequential Bonferroni correction.

Genetic distances based on Cavalli-Sforza's chord measure (*D_C_*) were computed using PHYLIP [41]. We used Poptree2 [42] to calculate Nei’s *D_A_* distance [24], Nei’s standard genetic distance (*D*_ST_) [25], and Latter’s *F*_ST_ * distance [26]. PCA was performed to visualize the patterns of the genetic relationships contained in *F*_ST_ and the four types of distances above using GenAlEx [43]. PCA is a process in which the major axes of variation are located within a multidimensional data set. For distinct groups, the first two or three axes will typically reveal most of the separation among them. A three-dimensional scatter chart was drawn to represent the PCA analysis result.

Population structure was investigated using STRUCTURE 2.3 [27]. STRUCTURE could not correctly identify the number of subpopulations at low levels of population differentiation (*F*_ST_<0.02) [44]. Hubisz et al. suggested that using the sampling location parameter of STRUCTURE (LOCPRIOR model) could partially overcome this limitation [45]. When the LOCPRIOR model is used, an *r* value is reported. An *r* value lower than 1 indicates that the sampling location in the model is effective [45]. We performed five runs for each cluster (*K*) from 2 to 9, using an MCMC chain with a burn-in length of 80,000 iterations, followed by 80,000 iterations under the LOCPRIOR model. Most of the parameters were set to their default values as advised in the user’s manual. Following the suggestion of Falush et al. [46], we allowed the degree of admixture α to be inferred from the data and chose the admixture model and the option of correlated allele frequencies between populations. True *K* was identified using the value of the average logarithmic probabilities across runs returned by STRUCTURE 2.3. The outputs from STRUCTURE were graphically modified by DISTRUCT [47].

Estimation of the admixture coefficient *mY* based on the average coalescence times between pairs of genes was evaluated by Admix 2.0. [28] When testing the northern origin hypothesis, we assumed that potential admixture began 2,500 years ago according to Dodd’s estimation. When testing the southern origin hypothesis, potential admixture was thought to have begun 2,000 years ago because, according to historical records, the Tai have lived in Yunnan for 2,000 years [3]. The mutation rate of dinucleotide microsatellites was set to 1.94×10^−4^ per generation [48], and a generation time of 25 years was used in all of the admixture analyses. Divergence of alleles models were not used in our admixture analyses because, by considering allele frequencies, the estimated coefficients are less affected by the stochasticity of the mutation process, and the timescale of the admixture process we wanted to investigate was comparable to the short times through which genetic drift acts, rather than to the long time that it takes for mutations to accumulate [49].

The simulation-based ABC analysis was performed using DIY-ABC v1.0.4.46b [29]. We constructed three scenarios according to the three hypotheses of the Tai origin (Figure 4). Pop 1 represents the Tai people in China (TC). Pop 2 represents the “Southern origin of the Tai” (ST), in which the Mulam and the Zhuang populations were pooled. Pop3 represents the “Northern origin of the Tai” (NT), in which the Mongolian, the Sala and the Dongxiang populations were pooled. Pop 4 represents the “Native origin of the Tai” (NAT), in which the Wa and the Bulang populations were pooled. Scenario 1 simulated what occurred in the northern origin hypothesis: the Tai people diverged from the people of northern China before t1 and migrated to southwestern China. The ancient Tai people admixed with the local people with a rate of r1. Scenario 2 simulated what occurred in the southern origin hypothesis: the Tai people diverged from the people of southern China before t1 and migrated to southwestern China. The ancient Tai people also admixed with local people with a rate of r1. Scenario 3 simulated the indigenous hypothesis: the Tai people simply diverged from the people of southwestern China. According to historical data, the separation occurred approximately 3,000 years ago (northern origin hypothesis) or 1,600 years ago (southern origin hypothesis). Thus, the boundary of t1 was set from 20 to 150 generations (a generation time of 25 years). Chu et al. [18] assumed that the ancestors of the Altaic-speaking populations were originally derived from Southeast Asia approximately 30,000 years ago, so the t3 boundary, which is the time the NT separated from the NAT, was set from 20 to 2,000 generations. Because we observed that the genetic distance between the ST and the NAT was small and there were no history records indicating that the separation of the ST and the NAT occurred within 3,000 years, we propose that the t2, which is the time the ST separated from the NAT, should be smaller than t3 and larger than t1 (t1<t2<t3). We chose flat prior distributions for all demographic parameters and listed their prior distributions for the ABC simulation in Table 3.

**Table 3:** The prior distributions of demographic parameters for the ABC simulation.

The prior distributions for the ABC simulation	Boundaries of these prior distributions
Effective population sizes of pooled population TC: *N1*	10 to 20,000
Effective population sizes of pooled population ST: *N2*	10 to 20,000
Effective population sizes of pooled population NT: *N3*	10 to 20,000
Effective population sizes of pooled population NAT: *N4*	10 to 20,000
The time after Tai separated from their parent population: *t1*	20 to 150 generations
The time after ST separated from the NAT: *t2*	20 to 2,000 generations; t2>t1
The time after NT separated from the NAT: *t3*	20 to 2,000 generations; t3>t2
The rate of Tai people admixed with NAT in SC1 and SC2: *r1*	0.01 to 0.99

Regarding the parameters for the microsatellite markers, each locus was assumed to follow a generalized stepwise mutation model (GSM) [50] with a possible range of 40 contiguous allelic states. The mean mutation rate was drawn in a uniform distribution that was bound between 10^−4^ and 10^−3^ [48] and excluded single-nucleotide insertions/deletions. Using these parameter values, we produced a reference table containing 1.5 million simulated data sets (500,000 for each scenario). Following Fagundes et al. [51], we performed a weighted polychotomous logistic regression to estimate the (relative) posterior probability of each scenario, taking the 15,000 simulated data sets (1%) closest to our actual data.

We evaluated the power of our methodology to discriminate between scenarios by analyzing test data sets simulated with identical numbers of loci and individuals as in our actual data set. Five hundred of such test data sets were simulated under each competing scenario, using parameter values drawn from the same prior distribution of those used in the ABC analyses. The posterior probabilities of each competing scenario were estimated for each simulated test data set and were used to compute type I and II errors in the choice of scenario by considering that the chosen scenario is the one with the highest probability value. We produced a total of 1,500 test data sets for the set of three competing scenarios that were analyzed to infer the evolutionary history of the Tai.

## Supporting Information

Table S1 **Standard diversity indices.** The allele frequencies, expected heterozygosities (*H*_E_) and Hardy-Weinberg Equilibrium (HWE) tests of the four new ethnic groups for the 10 microsatellites.
(XLS)

Table S2 **Genotypic data of 19 populations.** The genotypic data of 10 microsatellite markers in 19 populations.
(XLS)

Table S3 ***F*** **_ST_ of 19 populations.** The pairwise *F*_ST_ values and their significance among the 19 populations.
(XLS)
